# Potential prognostic value of *PD-L1* and *NKG2A* expression in Indonesian patients with skin nodular melanoma

**DOI:** 10.1186/s13104-021-05623-7

**Published:** 2021-05-28

**Authors:** Ridwan Dwi Saputro, Hanggoro Tri Rinonce, Yayuk Iramawasita, Muhammad Rasyid Ridho, Maria Fransiska Pudjohartono, Sumadi Lukman Anwar, Kunto Setiaji, Teguh Aryandono

**Affiliations:** 1 Department of Surgery, Faculty of Medicine, Public Health and Nursing, Universitas Gadjah Mada/Dr. Sardjito Hospital , Sleman, Yogyakarta, Indonesia; 2 Department of Anatomical Pathology, Faculty of Medicine, Public Health and Nursing, Universitas Gadjah Mada/Dr. Sardjito Hospital , Sleman , Yogyakarta, Indonesia

**Keywords:** *PD-L1*, *NKG2A*, Melanoma, Skin cancer, Indonesia

## Abstract

**Objective:**

Biomarker mRNA levels have been suggested to be predictors of patient survival and therapy response in melanoma cases. This study aimed to investigate the correlations between the mRNA expression levels of *PD-L1* and *NKG2A* in melanoma tissue with clinicopathologic characteristics and survival in Indonesian primary nodular melanoma patients.

**Results:**

Thirty-one tissue samples were obtained; two were excluded from survival analysis due to Breslow depth of less than 4 mm. The median survival of upregulated and normoregulated *PD-L1*-patients were 15.800 ± 2.345 and 28.945 ± 4.126 months, respectively. However, this difference was not significant statistically (*p* = 0.086). Upregulated and normoregulated NKG2A patients differed very little in median survival time (25.943 ± 7.415 vs 26.470 ± 3.854 months*; p* = 0.981). Expression of *PD-L1* and *NKG2A* were strongly correlated (r_s_: 0.787, *p* < 0.001). No clinicopathologic associations with *PD-L1* and *NKG2A* mRNA levels were observed. These results suggest that PD-L1 may have potential as a prognostic factor. Although an unlikely prognostic factor, *NKG2A* may become an adjunct target for therapy. The strong correlation between *PD-L1* and *NKG2A* suggests that anti-*PD-1* and anti-*NKG2A* agents could be effective in patients with *PD-L1* upregulation. The mRNA levels of these two genes may help direct choice of immunotherapy and predict patient outcomes.

**Supplementary Information:**

The online version contains supplementary material available at 10.1186/s13104-021-05623-7.

## Introduction

Among cutaneous malignancies, melanoma results in the highest mortality; statistics revealed that melanomas caused 287,723 new cases and 60,712 deaths worldwide in 2018 [1]. These numbers are expected to increase, with an estimated 340,721 new cases projected in 2025 [2]. Trials of various systemic chemotherapy combinations did not improve survival significantly. Thus, current research is focused on new therapeutic agents, including immune checkpoint blockers.

Agents that block immune checkpoints, such as *PD-1*/*PD-L1*, help improve the immune response to cancerous cells. Nivolumab, an anti-*PD-1* antibody, has been approved by the FDA for the treatment of advanced melanomas [3]. Other immunotherapies, such as monalizumab, a humanized anti-*NKG2A* antibody that enhances NK cell and CD8+ T cell activity [4], are also under development. These drugs show great promise and more durable responses compared with targeted therapy agents.

Before starting anti-*PD-1* immunotherapy, clinicians commonly test tumor tissues for *PD-L1* expression by using immunohistochemistry (IHC). Tumors expressing *PD-L1* respond better to anti-*PD-1*/*PD-L1* agents compared with non-expressers [5]. However, the results of recent studies on the effect of *PD-L1* expression on survival are conflicting [6]. Detection using IHC also presents several limitations, such as the varying performance of different antibodies, nonstandard cut-off values, and operator dependence [7, 8].

Researchers are exploring new methods to predict therapy responses and survival in melanoma patients. Several studies have investigated the use of biomarker mRNA levels as an alternative parameter [9–11]. Gupta et al*.* reported that mRNA levels of *PD-L1*/2 show potential in predicting survival and response toward immunotherapy in metastatic melanoma [12]. Given the emergence of monalizumab, the potential prognostic and therapeutic roles of *NKG2A* should also be investigated.

Immuno-oncological research is still rare in Indonesia. Most Indonesian patients are treated with surgical resection, dacarbazine chemotherapy, and radiotherapy. Information on the expression of immune-checkpoint molecules is needed to gauge the potential efficacy of using immunotherapy agents in Indonesia. Thus, this study aimed to investigate the prognostic role of mRNA levels of *PD-L1* and *NKG2A*, as well as the associated clinicopathologic characteristics.

## Main text

### Materials and methods

Formalin-fixed paraffin-embedded (FFPE) tissue samples from patients diagnosed in 2012–2019 with primary cutaneous nodular melanoma were collected from the archives of the Department of Anatomical Pathology, Dr. Sardjito Hospital, which is the main cancer referral center in Yogyakarta, Indonesia. Cases with prior chemotherapy or radiotherapy, incomplete clinical data, and degraded specimens were excluded. Thirty-one samples were analyzed in this retrospective cohort study, and all patients were of Javanese ethnicity.

RNA was extracted from FFPE tissues using GeneAll® Ribospin™ II (GeneAll Biotechnology, Seoul, South Korea). Real-time polymerase chain reaction (RT-PCR) for *PD-L1* and *NKG2A* expression quantification was conducted using AccuPower® GreenStar™ RT-qPCR PreMix on an Exicycler™ 96 (Bioneer Corp., Daejeon, South Korea) with primer pairs and thermocycler conditions as previously described by Vassilakopoulou et al*.* and Meckawy et al*.* [13, 14]. The expressions of *PD-L1* and *NKG2A* were calculated from the quantification cycle (Cq) values of the gene targets and normalized against *GAPDH* as an internal control. Subsequent normalization was performed using the ΔΔCq values of RNA derived from healthy skin tissues. Age, sex, tumor location, Breslow thickness, greatest diameter, lymph node involvement, and stage were retrieved from medical records. Pathological data, including the presence of necrosis, lymphovascular invasion, tumor-infiltrating lymphocytes (TILs), and mitotic index, were obtained from hematoxylin–eosin and Ki-67 IHC stained slides. Survival status (living or deceased) was determined through telephone calls at the point of follow-up of the study (until April 2020).

Samples were classified as normoregulated if the expression was lower than or equal to the mean of the *PD-L1* and *NKG2A* levels; conversely, samples were classified as upregulated if the expression was above the mean. Comparison of mRNA level averages based on categorical clinicopathologic characteristics was performed using Mann–Whitney U tests. Spearman correlation was used to analyze associations between the expression of *PD-L1* and *NKG2A* and continuous clinicopathologic features. Kaplan–Meier analysis and log-rank tests with Cox regression were used to determine hazard ratios (HRs) for survival analysis. To minimize the effect from Breslow thickness, two samples was excluded from survival analysis due to depth of less than 4 mm.

### Results

The characteristics of the subjects are presented in Table 1. Most tumors were located on the extremities (70.97%) and thicker than 4 mm (93.55%). Necrosis and TILs were present in 74.19% of the samples, respectively. The clinical stages were evenly distributed among stages II (29.03%), III (35.48%), and IV (35.48%).

**Table 1:** Clinicopathologic characteristics of the subjects

Age (years), mean ± SD	61.68 ± 16.54
Sex, n (%)	
Male	8 (25.81)
Female	23 (74.19)
Tumor location, n (%)	
Trunk	2 (6.45)
Head and neck	7 (22.58)
Extremity	22 (70.97)
Lymph node metastases, n (%)	
Present	21 (67.74)
Absent	10 (32.26)
Breslow thickness	
≤ 4.00 mm	2 (6.45)
> 4.00 mm	29 (93.55)
Necrosis, n (%)	
Present	23 (74.19)
Absent	8 (25.81)
Tumor diameter (mm), mean ± SD	30.00 ± 24.09
Ulceration	
Present	16 (51.61)
Absent	15 (48.39)
Tumor-infiltrating lymphocytes	
Present (brisk and non-brisk)	23 (74.19)
Absent	8 (25.81)
Clinical stage	
I	0 (0.00)
II	9 (29.03)
III	11 (35.48)
IV	11 (35.48)
Survival status	
Alive	7 (22.58)
Deceased	24 (77.42)
Overall survival (months), mean ± SD	22.84 ± 15.75

SD: standard deviation

The expression of *PD-L1* and *NKG2A* was not significantly associated with the patients’ clinicopathologic characteristics (Additional file 1: Table S1). Spearman correlation showed that *NKG2A* and *PD-L1* mRNA levels were strongly correlated (Additional file 2: Table S2).

In the Cox univariate regression analysis, higher stage, upregulated *PD-L1*, and upregulated *NKG2A* were related to higher risks of death, with respective HR of 1.080 (*p* = 0.763), 2.429 (*p* = 0.101), and 1.011 (*p* = 0.981) (Additional file 3: Table S3). In multivariate analysis, the HR for *PD-L1* increased to 3.488 (*p* = 0.066), while the other HRs decreased to 1.017 (*p* = 0.951) for stage and 0.590 (*p* = 0.391) for *NKG2A* upregulation. However, the differences were not statistically significant.

**Fig. 1:**
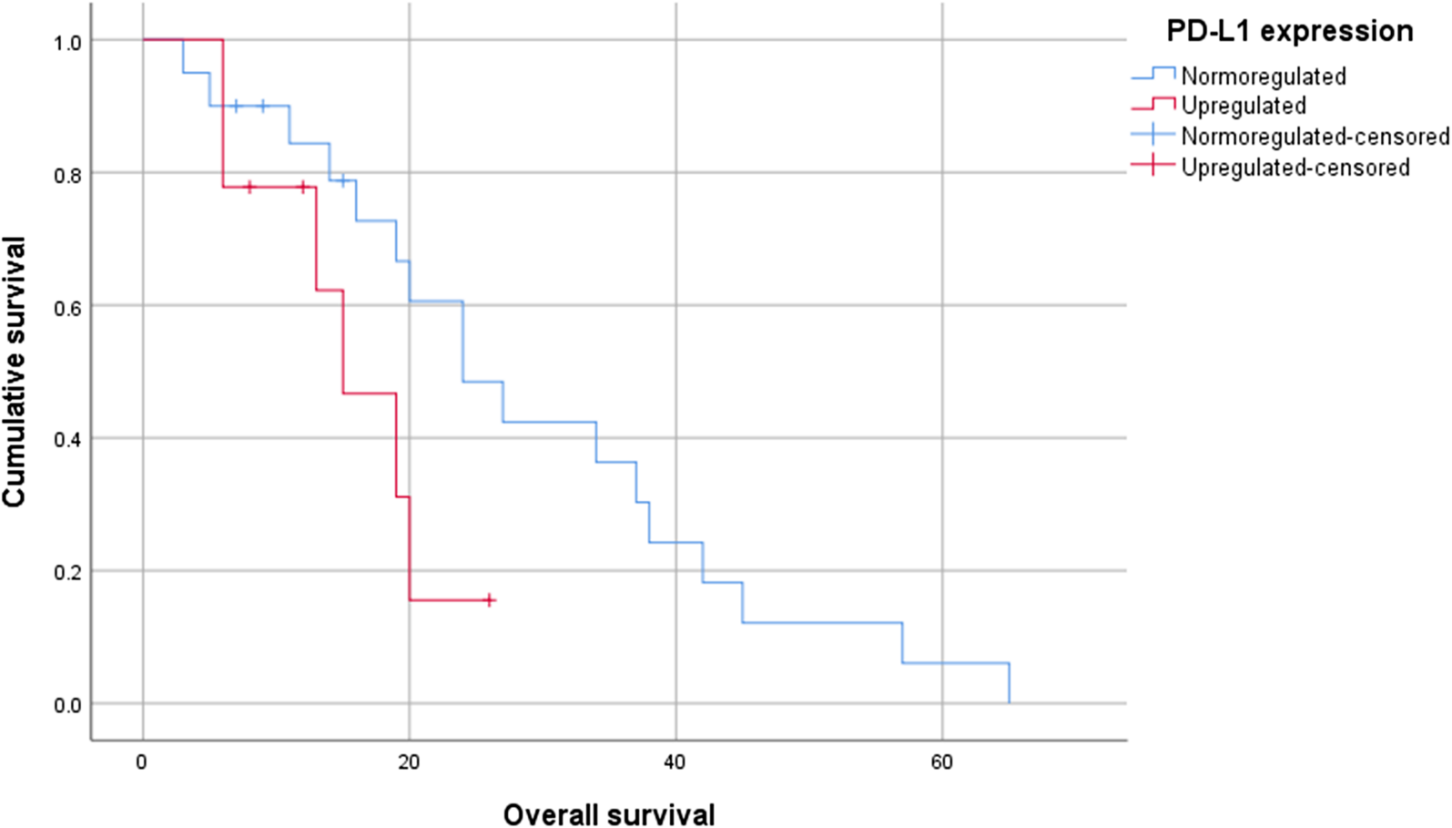
Kaplan–Meier survival curves comparing the survival of patients with primary nodular melanoma with upregulated or normoregulated *PD-L1* expression (*p* = 0.086)

**Fig. 2:**
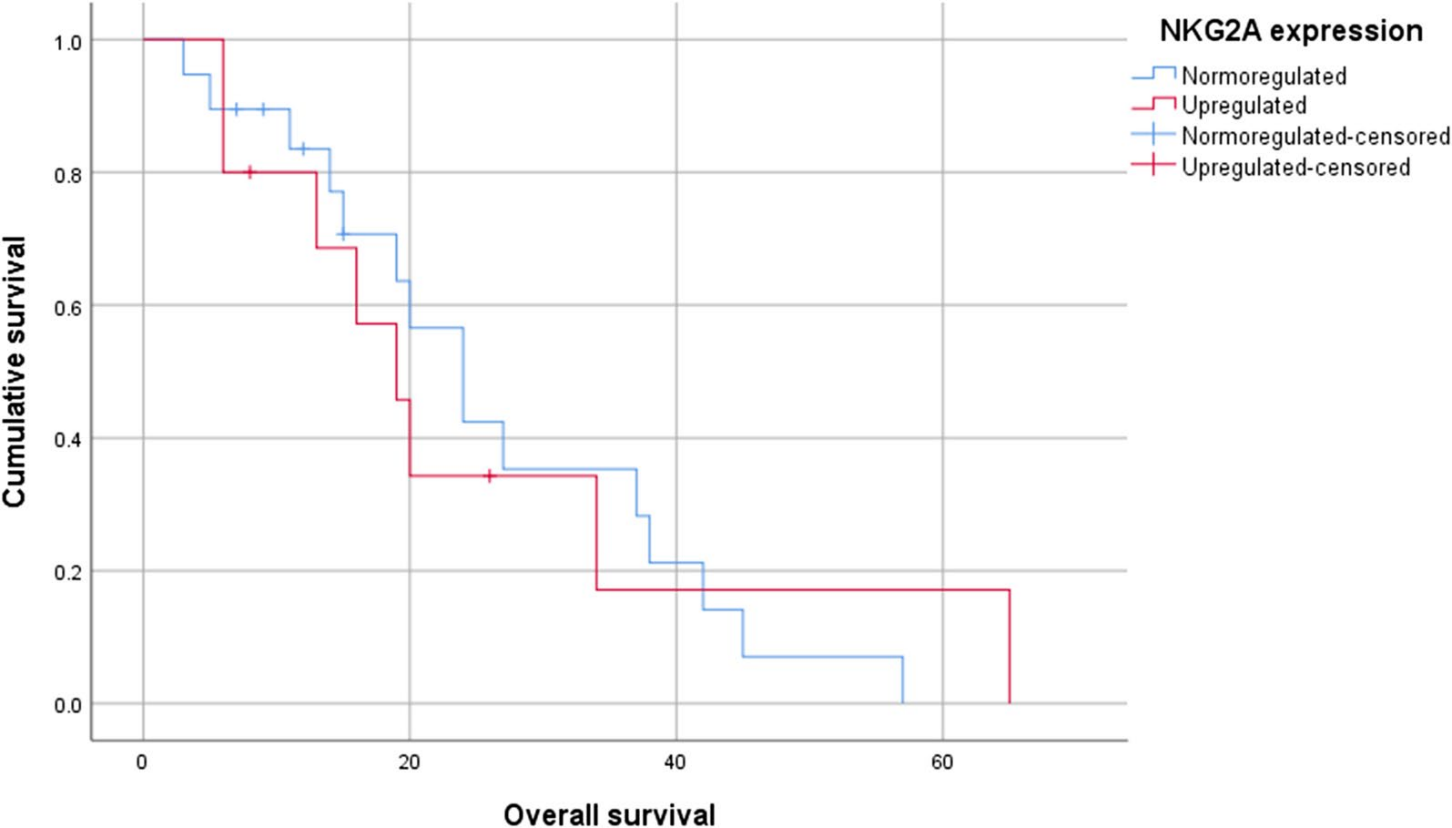
Kaplan–Meier survival curves comparing the survival of patients with primary nodular melanoma with upregulated or normoregulated *NKG2A* expression (*p* = 0.981)

Patients with normoregulated *PD-L1* expression had longer median survival time (28.945 ± 4.126 months) compared with upregulated expressers (15.800 ± 2.345 months; *p* = 0.086) (Fig. 1). Similar findings were observed for the normoregulated (26.470 ± 3.854 months) and upregulated *NKG2A* subjects (25.943 ± 7.415 months*; p* = 0.981) (Fig. 2). However, both differences were not significant statistically. Presence of TILs did not affect the survival curves significantly (*p* = 0.422) (Additional file 4: Fig. S1). The survival curves of the upregulated and normoregulated groups for *PD-L1* and *NKG2A* did not differ significantly when divided based on the presence of TILs (Additional file 5: Fig. S2 and Additional file 6: Fig. S3).

### Discussion

In this study, we compared the clinicopathologic characteristics and overall survival of Indonesian primary nodular melanoma cases with different mRNA levels of *PD-L1* and *NKG2A*. Two important findings were observed: (1) cases with *PD-L1* overexpression tended to have lower survival rates and (2) *PD-L1* and *NKG2A* levels were strongly correlated.

Melanoma patients with upregulated *PD-L1* tended to have lower overall survival, with an approximately two to threefold higher HR compared to normal expressers. This trend was not proven statistically significant, possibly due to the small sample size. However, the increase in the HR for *PD-L1* upregulation in multivariate regression suggests this trend may exist in the overall population of melanoma patients in Indonesia.

The *PD-L1* molecule interacts with *PD-1* receptors on T cells, causing anergy, exhaustion, and apoptosis [15]. Melanoma cells can thus evade the immune system by increasing their *PD-L1* expression. Melanoma cell line studies show that cells with upregulated *PD-L1* demonstrate highly invasive and aggressive behavior [16]. In a study on melanoma patients treated with surgery and dacarbazine, patients with positive *PD-L1* on IHC staining had lower median survival time compared with the subgroup with negative or indeterminate *PD-L1* status (9.7 months vs. 11.6 months) [17].

Our results seem to oppose those of Gupta et al*.*, who observed that higher *PD-L1* mRNA levels reflect better prognoses for melanoma patients treated with anti-*PD-1* agents [12]. This disparity may have stemmed from differences in the treatment regimen. Patients with high expression of *PD-L1* respond well to anti-*PD-1* antibodies, hence the increase in survival [5]. The results suggest that *PD-L1* expression is a negative prognostic factor in patients with melanoma treated with conventional chemotherapy. However, when treated with anti-*PD-1* antibodies, patients with high levels of *PD-L1* respond well and have good outcomes. Therefore, the choice of therapy also affects the performance of *PD-L1* as a prognostic factor.

Tumor cells can express *PD-L1* independently (called constitutive expression) or in response to TILs (reactive expression). TILs can secrete interferon-gamma, which induces the expression of *PD-L1* in tumor cells [15]. When *PD-L1* expression is accompanied by the presence of TILs, the expression is likely reactive, and vice versa. Due to the strongly positive correlation between *PD-L1* and TILs, studies report that most melanomas express *PD-L1* reactively [18]. However, in our study, the proportion of upregulated PD-L1 among the cases without TILs was higher (37.5%) than the cases with TILs (25%). The groups without TILs also had higher average *PD-L1* mRNA levels than the group with TILs. This finding suggests that the cases with upregulated *PD-L1* in our study are likely constitutive expressers.

These two modes of *PD-L1* expression may have different prognostic implications. When previous studies divided patients based on *PD-L1* expression and the presence of TILs, patients with constitutive *PD-L1* expression without lymphocyte infiltrates showed the poorest outcomes, followed by reactive *PD-L1* expressers, those with *PD-L1*(−) without TILs, and, finally, those with *PD-L1*(−) and TILs [16, 19]. In our results, both groups with upregulated *PD-L1* showed poorer prognoses than the groups with normoregulated *PD-L1*. However, survival did not differ significantly when the cases were divided further based on TIL status, likely because none of the patients were treated using immunotherapy, in which the presence of TILs predicts improved response [20]. Another factor that could explain this lack of significance is the limited sample size.

Upregulated *NKG2A* mRNA did not appear to affect survival, given the highly insignificant results in univariate and multivariate regression analysis. *NKG2A* is an inhibitory receptor found on NK cells [21]. Cancer cells can attempt to evade the immune system by upregulating HLA self-molecules that activate *NKG2A* receptors and impair the function of NK cells. Trials in mouse models indicate that monalizumab is ineffective as a single therapy but highly effective when used together with other immunotherapy agents that promote activated TILs, such as anti-*PD-1* or cancer vaccines [22]. One escape strategy used by cells to escape cytotoxic TILs is downregulation of MHC I expression, which renders them targets for NK cells [23]. This finding may explain the role of anti-*NKG2A* as an adjunct treatment for other immunotherapies. Our results reinforce the idea that *NKG2A* may not be an independent therapeutic target and prognostic factor but may play a role in combination therapy.

The mRNA expressions of *NKG2A* and *PD-L1* were strongly correlated. This finding indicates that tumors with high *PD-L1* expression would also likely express *NKG2A* strongly and, thus, respond well to anti-*NKG2A* agents. When *NKG2A* expression was combined with the TIL parameter, the distribution of survival curves obtained resembled the curves for *PD-L1* combined with TILs. *NKG2A* upregulation with and without the presence of TILs may have different pathogeneses and prognostic implications, like *PD-L1*.

The lack of correlation between the expression of *NKG2A* and *PD-L1* and clinicopathologic characteristics in this work resembles the findings of several previous studies [6, 18]. The small sample size of this study may have contributed to the low statistical significance found.

The findings of this study must be interpreted with caution especially due to the small sample size. However, our results support the findings of several studies that show that mRNA profiles may serve as a prognostic factor in melanoma cases [12, 24]. Further research and clinical trials are needed to ascertain the roles of *PD-L1* and *NKG2A* in the prognosis and therapy of Asian patients who have not previously received immune checkpoint inhibitors.

### Conclusions

We investigated the correlations between the mRNA levels of *PD-L1* and *NKG2A* with clinicopathologic characteristics and survival in primary nodular melanoma patients in Yogyakarta, Indonesia. Patients with upregulated *PD-L1* expression had shorter median overall survival, although insignificant statistically. *PD-L1* and *NKG2A* mRNA levels were positively correlated.

Our findings suggest that the therapy regimen and presence of TILs may affect the prognostic role of *PD-L1* expression. *NKG2A* was not proven to be an independent predictive factor but may serve as an adjunct target for therapy. The strong correlation between *PD-L1* and *NKG2A* suggests that anti-*PD1* and anti-*NKG2A* agents may be effective in patients with *PD-L1* upregulation. Studies with larger subject groups are needed to confirm the patterns of *PD-L1* expression in Asian cases.

## Limitations

Our study was limited by its small sample size and homogenous ethnic population. Results among diverse Indonesian and Asian populations may differ. TILs examination did not discriminate between lymphocyte subtypes.

## Supplementary Information

**Additional file 1: Table S1.** Comparison of *PD-L1* and *NKG2A* expression levels based on clinicopathologic characteristics and overall survival.

**Additional file 2: Table S2.** Spearman correlations between continuous variables.

**Additional file 3: Table S3.** Univariate and multivariate Cox regression results for stage, *PD-L1* upregulation, and *NKG2A* upregulation.

**Additional file 4: Fig. S1.** Kaplan–Meier survival curves comparing the survival of patients with primary nodular melanoma with or without TILs.

**Additional file 5: Fig. S2.** Kaplan–Meier survival curves comparing the survival of patients with primary nodular melanoma based on *PD-L1* expression and the presence of TILs.

**Additional file 6: Fig. S3.** Kaplan–Meier survival curves comparing the survival of patients with primary nodular melanoma based on *NKG2A* expression and the presence of TILs.

## Data Availability

This submission contains all of the data analyzed during the study. Unprocessed data can be requested from the corresponding author.

## Abbreviations

CD: Cluster of differentiation
Cq: Quantification cycle
DNA: Deoxyribonucleic acid
FFPE: Formalin-fixed paraffin-embedded
GADPH: Glyceraldehyde 3-phosphate dehydrogenase
HLA: Human leukocyte antigen
IHC: Immunohistochemistry
MHC: Major histocompatibility complex
mRNA: Messenger ribonucleic acid
NK: Natural killer
NKG2A: Natural killer group 2A
PD-1: Programmed death-1
PD-L1: Programmed death-ligand 1
RT-PCR: Real-time polymerase chain reaction
TILs: Tumor-infiltrating lymphocytes
