# Supplementary material for: Potential prognostic value of PD-L1 and NKG2A expression in Indonesian patients with skin nodular melanoma
Source: BMC Res Notes. 2021 May 28;14:206. doi: 10.1186/s13104-021-05623-7 (PMC8161664; doi:10.1186/s13104-021-05623-7)
Supplement: Supplementary file 1 — Additional file 1: Table S1. Comparison of PD-L1 and NKG2A expression levels based on clinicopathologic characteristics and overall survival. [file 13104_2021_5623_MOESM1_ESM.docx]

**Table S1** Comparison of *PD-L1* and *NKG2A* expression levels based on clinicopathologic characteristics and overall survival

| Clinicopathologic characteristics | *PD-L1* Expression | | *p* value | *NKG2A* Expression | | *p* value |  |
| --- | --- | --- | --- | --- | --- | --- | --- |
|  | n | Average |  | n | Average |  |  |
| Age |  |  |  |  |  |  |  |
| <60 years | 8 | 7.96 | 0.503 | 8 | 3.16 | 0.322 |  |
| ≥60 years | 23 | 9.19 |  | 23 | 5.67 |  |  |
| Sex |  |  |  |  |  |  |  |
| Male | 8 | 8.50 | 0.784 | 8 | 7.19 | 0.249 |  |
| Female | 23 | 9.00 |  | 23 | 4.27 |  |  |
| Location |  |  |  |  |  |  |  |
| Extremity | 22 | 9.13 | 0.616 | 22 | 4.98 | 0.951 |  |
| Central | 9 | 8.24 |  | 9 | 5.13 |  |  |
| Diameter |  |  |  |  |  |  |  |
| ≤4 mm | 3 | 4.83 | 0.094 | 3 | 2.83 | 0.521 |  |
| >4 mm | 28 | 9.31 |  | 28 | 5.25 |  |  |
| Breslow thickness |  |  |  |  |  |  |  |
| ≤4 mm | 2 | 7.12 | 0.567 | 2 | 2.87 | 0.614 |  |
| >4 mm | 29 | 8.99 |  | 29 | 5.17 |  |  |
| Ulceration |  |  |  |  |  |  |  |
| Absent | 15 | 9.42 | 0.514 | 15 | 6.88 | 0.099 |  |
| Present | 16 | 8.36 |  | 16 | 3.27 |  |  |
| Necrosis |  |  |  |  |  |  |  |
| Absent | 8 | 9.48 | 0.659 | 8 | 6.98 | 0.298 |  |
| Present | 23 | 8.66 |  | 23 | 4.34 |  |  |
| Lymphovascular invasion | | | | | | | |
| Absent | 22 | 8.72 | 0.764 | 22 | 5.77 | 0.289 |  |
| Present | 9 | 9.25 |  | 9 | 3.18 |  |  |
| Lymph node involvement | | | | | | | |
| Absent | 10 | 8.53 | 0.770 | 10 | 3.77 | 0.439 |  |
| Present | 21 | 9.04 |  | 21 | 5.62 |  |  |
| Tumor-infiltrating lymphocyte | | | | | | | |
| Absent | 8 | 10.48 | 0.236 | 8 | 4.62 | 0.834 |  |
| Present | 23 | 8.31 |  | 23 | 5.16 |  |  |
| Clinical stage |  |  |  |  |  |  |  |
| Stage I–III | 20 | 8.86 | 0.925 | 20 | 3.79 | 0.268 |  |
| Stage IV | 11 | 8.90 |  | 11 | 7.26 |  |  |
| Overall survival |  |  |  |  |  |  |  |
| <12 months | 9 | 8.93 | 0.964 | 9 | 3.70 | 0.450 |  |
| ≥12 months | 22 | 8.85 |  | 22 | 5.56 |  |  |

**p* value < 0.05 was considered significant
