# Supplementary material for: Potential prognostic value of PD-L1 and NKG2A expression in Indonesian patients with skin nodular melanoma
Source: BMC Res Notes. 2021 May 28;14:206. doi: 10.1186/s13104-021-05623-7 (PMC8161664; doi:10.1186/s13104-021-05623-7)
Supplement: Supplementary file 2 — Additional file 2: Table S2. Spearman correlations between continuous variables. [file 13104_2021_5623_MOESM2_ESM.docx]

**Table S2** Spearman correlations between continuous variables

|  | Age | Mitotic index | Size | Stage | Overall survival | *NKG2A* | *PD-L1* |
| --- | --- | --- | --- | --- | --- | --- | --- |
| Age |  | 0.180 (0.333) | 0.359 (**0.047**) | −0.100 (0.592) | −0.225 (0.223) | 0.090 (0.631) | −0.043 (0.817) |
| Mitotic index |  |  | 0.079 (0.675) | 0.075 (0.688) | −0.137 (0.463) | 0.053 (0.776) | −0.029 (0.877) |
| Size |  |  |  | −0.149 (0.425) | −0.304 (0.096) | 0.057 (0.761) | 0.270 (0.142) |
| Stage |  |  |  |  | 0.088 (0.636) | −0.117 (0.531) | −0.065 (0.729) |
| Overall survival |  |  |  |  |  | −0.086 (0.644) | −0.038 (0.838) |
| *NKG2A* |  |  |  |  |  |  | 0.787 (**0.000**) |
| *PD-L1* |  |  |  |  |  |  |  |

**p* value < 0.05 was considered significant
