# Supplementary material for: Potential prognostic value of PD-L1 and NKG2A expression in Indonesian patients with skin nodular melanoma
Source: BMC Res Notes. 2021 May 28;14:206. doi: 10.1186/s13104-021-05623-7 (PMC8161664; doi:10.1186/s13104-021-05623-7)
Supplement: Supplementary file 3 — Additional file 3: Table S3. Univariate and multivariate Cox regression results for stage, PD-L1 upregulation, and NKG2A upregulation. [file 13104_2021_5623_MOESM3_ESM.docx]

**Table S3** Univariate and multivariate Cox regression results for stage, *PD-L1* upregulation, and *NKG2A* upregulation

|  | (Univariate) HR (95% CI) | *p* value | (Multivariate) HR  (95% CI) | *p* value |
| --- | --- | --- | --- | --- |
| Stage | 1.080  (0.654–1.783) | 0.763 | 1.017  (0.602–1.717) | 0.951 |
| *PD-L1* | 2.429  (0.841–7.021) | 0.101 | 3.488  (0.920–13.221) | 0.066 |
| *NKG2A* | 1.011  (0.408–2.503) | 0.981 | 0.590  (0.177–1.970) | 0.391 |

**p* value < 0.05 was considered significant; CI, confidence interval; HR, hazard ratio
