# Supplementary material for: Potential prognostic value of PD-L1 and NKG2A expression in Indonesian patients with skin nodular melanoma
Source: BMC Res Notes. 2021 May 28;14:206. doi: 10.1186/s13104-021-05623-7 (PMC8161664; doi:10.1186/s13104-021-05623-7)
Supplement: Supplementary file 4 — Additional file 4: Fig. S1. Kaplan–Meier survival curves comparing the survival of patients with primary nodular melanoma with or without TILs. [file 13104_2021_5623_MOESM4_ESM.docx]

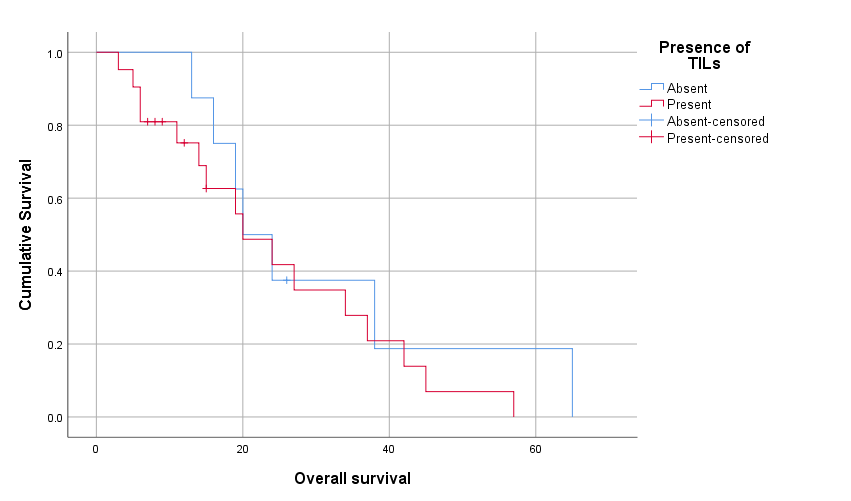


**Fig. S1** Kaplan–Meier survival curves comparing the survival of patients with primary nodular melanoma with or without TILs (*p* = 0.422)
