# Supplementary material for: Potential prognostic value of PD-L1 and NKG2A expression in Indonesian patients with skin nodular melanoma
Source: BMC Res Notes. 2021 May 28;14:206. doi: 10.1186/s13104-021-05623-7 (PMC8161664; doi:10.1186/s13104-021-05623-7)
Supplement: Supplementary file 5 — Additional file 5: Fig. S2. Kaplan–Meier survival curves comparing the survival of patients with primary nodular melanoma based on PD-L1 expression and the presence of TILs. [file 13104_2021_5623_MOESM5_ESM.docx]

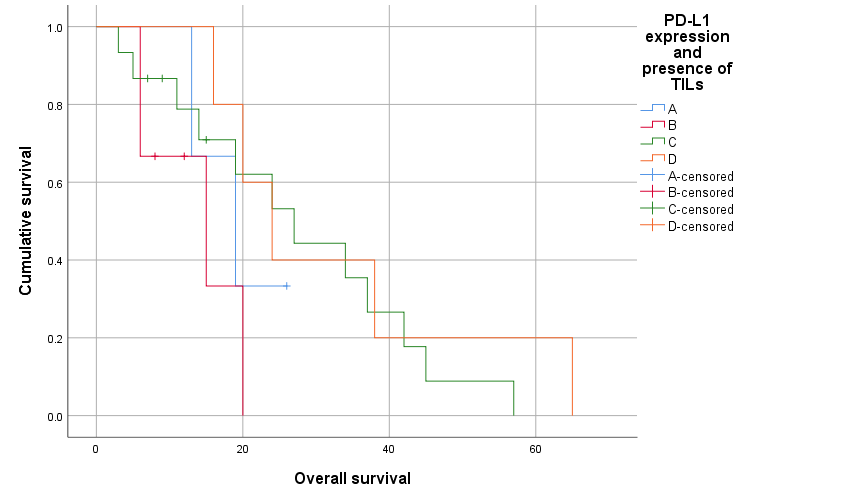


**Fig. S2** Kaplan–Meier survival curves comparing the survival of patients with primary nodular melanoma based on *PD-L1* expression and the presence of TILs (*p* = 0.196)

(A: upregulated *PD-L1* without TILs, B: upregulated *PD-L1* with TILs,

C: normoregulated *PD-L1* with TILs, D: normoregulated *PD-L1* without TILs)
